# Transcranial focused ultrasound stimulation of human primary visual cortex

**DOI:** 10.1038/srep34026

**Published:** 2016-09-23

**Authors:** Wonhye Lee, Hyun-Chul Kim, Yujin Jung, Yong An Chung, In-Uk Song, Jong-Hwan Lee, Seung-Schik Yoo

**Affiliations:** 1 Incheon St. Mary’s Hospital, The Catholic University of Korea, Incheon, Korea; 2 Department of Radiology, Brigham and Women’s Hospital, Harvard Medical School, Boston, MA, USA; 3 Department of Brain and Cognitive Engineering, Korea University, Seoul, Korea

## Abstract

Transcranial focused ultrasound (FUS) is making progress as a new non-invasive mode of regional brain stimulation. Current evidence of FUS-mediated neurostimulation for humans has been limited to the observation of subjective sensory manifestations and electrophysiological responses, thus warranting the identification of stimulated brain regions. Here, we report FUS sonication of the primary visual cortex (V1) in humans, resulting in elicited activation not only from the sonicated brain area, but also from the network of regions involved in visual and higher-order cognitive processes (as revealed by simultaneous acquisition of blood-oxygenation-level-dependent functional magnetic resonance imaging). Accompanying phosphene perception was also reported. The electroencephalo graphic (EEG) responses showed distinct peaks associated with the stimulation. None of the participants showed any adverse effects from the sonication based on neuroimaging and neurological examinations. Retrospective numerical simulation of the acoustic profile showed the presence of individual variability in terms of the location and intensity of the acoustic focus. With exquisite spatial selectivity and capability for depth penetration, FUS may confer a unique utility in providing non-invasive stimulation of region-specific brain circuits for neuroscientific and therapeutic applications.

Non-invasive brain stimulation methods, such as transcranial magnetic stimulation (TMS) and transcranial direct current stimulation (tDCS), modulate the function of cortical areas without surgical interventions^1^,^2^; however, the size of the modulatory area is rather large (on the order of centimeters) while ability to reach specific regions in deep cortical/subcortical areas is limited^1^,^3^. Accordingly, more spatially-selective means for non-invasive brain stimulation that can also reach deep brain areas have been long sought after.

Advancements in FUS technology enable the transcranial delivery of the acoustic energy to highly-localized areas (on the order of a few millimeters) across the brain, including deep brain structures^4^,^5^,^6^. This ability has been utilized to thermally ablate a specific brain region using high acoustic intensity in the context of functional neurosurgery^4^,^6^. Meanwhile, *in vitro*^7^,^8^ and *in vivo*^9^,^10^,^11^,^12^,^13^ studies have shown that acoustic pressure waves, given at a low intensity that does not cause temperature elevation, can modulate the excitability of the sonicated region of the brain with excellent spatial selectivity. Additional evidence from large animals showed that FUS can elicit electrophysiological responses by stimulating the visual and motor cortices in sheep^14^ and can functionally modulate the behavioral responses (*e.g.*, saccadic movement) by stimulating the frontal eye field in non-human primates^15^.

In humans, transcranial application of FUS has shown to temporarily change the activity of the primary somatosensory area (SI), as manifested through the modulation of tactile discrimination task performance and somatosensory evoked potentials (SEP) generated by median nerve stimulation^16^. More recently, elicitation of tactile sensations and associated evoked EEG potentials, in the absence of external sensory stimulation, was observed during the FUS stimulation of the SI^17^. With its superior spatial selectivity and its feasibility for use in humans, transcranial FUS is gaining momentum as a new mode of non-invasive brain stimulation that may provide new opportunities in the assessment of normal and aberrant region-specific brain functions as well as in the development of various modes of neurotherapeutics.

Concurrent functional assessment of neural circuits in the brain during FUS-mediated neuromodulation would provide a basis for advancing our knowledge of acoustic brain stimulation. Functional neuroimaging modalities, such as functional magnetic resonance imaging (fMRI) and positron emission tomography (PET), have been used to characterize the spatial and temporal features of neural responses to FUS-mediated brain stimulation in anesthetized small animals^13^,^18^,^19^. Yet, evidence of increased neural activity in the stimulated region, as well as the associated network-wide brain responses, remains unexamined in conscious humans. In the present study, we delivered stimulatory FUS to the primary visual cortex (V1) in humans as guided by the functional neuroanatomy of the individuals. fMRI was acquired simultaneously to identify the brain areas that were activated due to the stimulation. The presence and the type of visual perception elicited by the stimulation were also probed. Separate from the fMRI evaluation, we also measured cortical EEG potentials associated with the FUS stimulation to provide an electrophysiological assessment of brain activity. Numerical simulation of acoustic wave propagation through the skull was performed retrospectively to estimate the on-site acoustic intensity and spatial accuracy of the sonication. Our findings suggest that FUS can stimulate the human V1, resulting in the perception of phosphene, while also activating a network of regions involved in visual and higher-order cognitive processes.

## Results

### Image-guided transcranial FUS to the primary visual cortex

**Figure 1:**
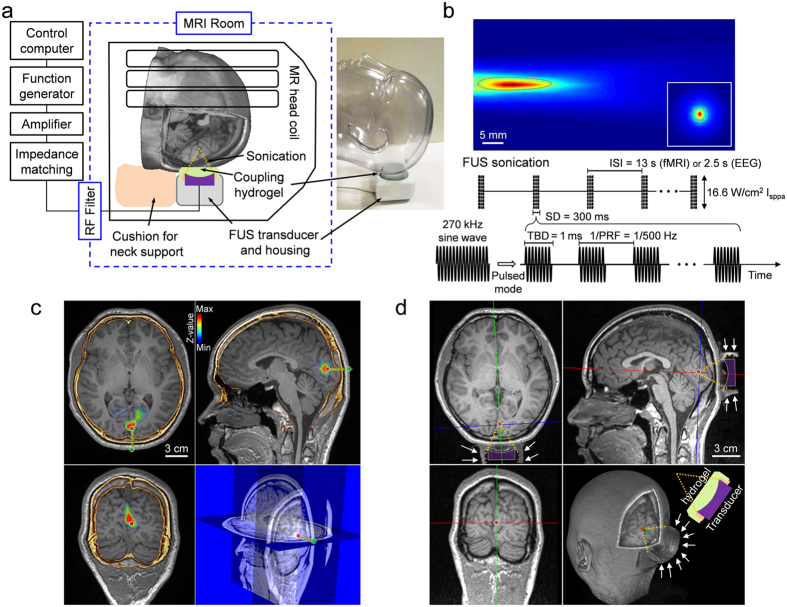
Schematics of the MR-guided transcranial FUS sonication setup and parameters. (**a**) The FUS apparatus positioned inside of the MR head coil, with the illustration of the sonication path (dotted yellow lines). A picture of the corresponding implementation is also shown with a mannequin head (without an MR head coil). (**b**) Upper panel: acoustic intensity profile (longitudinal and transversal, inset) of the FUS focus generated by the transducer. The longitudinal profile was measured with a 20 mm gap from the exit plane of the transducer. A cigar-shaped (3 mm in diameter and 17 mm in length) acoustic focus based on the full-width at half-maximum (FWHM) of the acoustic intensity is depicted by the dashed red line. Scale bar, 5 mm. Lower panel: illustration of the acoustic parameters. ISI, inter-stimulation-interval = 13 s in fMRI sessions. 2.5 s in EEG sessions; TBD, tone-burst-duration; PRF, pulse-repetition-frequency; Incident spatial-peak pulse-average acoustic intensity = 16.6 W/cm^2^ I_sppa_. (**c**) Exemplar anatomical MR images overlaid with the functional MRI (*P* < 0.005, uncorrected; *t*-value map in pseudo color) and cranial CT (marked in yellow/orange), which were used to plan the sonication path (green line) and the entry point (green dot on the scalp surface) to reach the target (red dot within a white circle). The right lower panel shows the corresponding information on the sectional MRI. (**d**) A snapshot of MRI (T1-weighted) taken during the MR-guided sonication session, whereby the profile of the acoustic coupling hydrogel, being compressed to ~1 cm thickness, is visible (white arrows). The geometric location of the sonication path with respect to the focal point (red dot) is illustrated with dotted yellow lines. The contour of the FUS transducer is demarcated in purple profile.

Head MRI (which included fMRI mapping of the visual areas) and computed tomography (CT) data were obtained from 19 volunteer participants (indexed ‘h1’–‘h19’) and were co-registered to provide the individual-specific V1 location for planning the administration of sonication. Then, in a separate experimental session, an MR-compatible FUS setup (experimental schematics depicted in Fig. 1a, see also Supplementary Methods) was used to transcranially deliver pulsed low-intensity FUS to the V1 under the guidance of a clinical 3-Tesla MR scanner. We used a 270 kHz frequency ultrasound to stimulate the V1, whereby similar ranges of frequencies have been employed to modulate human/ovine brain activity^14^,^17^ and to achieve adequate transcranial transmission through the skull^20^. The focal length of the FUS transducer was 3 cm, and the size of the acoustic focus was measured to be 3 mm in diameter and 17 mm in length, as defined by the full-width at half-maximum (FWHM) of the acoustic intensity map (Fig. 1b, upper panel). We used a similar pulsing scheme that had previously resulted in the successful stimulation of the SI in humans^17^ and animals^10^,^14^: sonication duration of 300 ms with a tone burst duration (TBD) of 1 ms repeated at a pulse repetition frequency (PRF) of 500 Hz (yielding a 50% duty cycle, illustrated in Fig. 1b, lower panel). The incident acoustic intensity and pressure at the FUS focus were 16.6 W/cm^2^ (spatial-peak pulse-average intensity, I_sppa_) and 1.48 MPa, respectively. After aligning the acoustic focus to the V1 in reference to the individual-specific functional data (from fMRI) and skull neuroanatomy (from CT) (example shown in Fig. 1c), FUS was applied to the V1 through a polyvinyl alcohol (PVA) hydrogel^21^, which was compressed to ~1 cm thickness around the contour of the skin (from its original thickness of 15 mm), achieved tight acoustic coupling while maintaining the orientation of the sonication entry as perpendicular as possible to the scalp (Fig. 1d).

### FUS stimulation of the V1 elicited phosphene perception

Event-related fMRI was simultaneously conducted during three different types of experimental conditions to capture the neural activity associated with (1) FUS, (2) sham FUS (*i.e.*, mimicking the FUS procedures but without sonication), and (3) photic stimulation without FUS, all while being synchronized with the MR scanner operation. The participants, blinded to the nature of the FUS stimulation, were asked to provide descriptions of any sensation using their own words after the real/sham FUS sessions. Out of 19 participants, 11 subjects (*N* = 11, ‘h1’–‘h11’) reported the perception of phosphene (defined as ‘responsive’ for categorical purposes) associated with real FUS stimulation while none reported any sensations during the sham FUS condition. Most of the visual perceptions were described as a diffuse, amorphous, non-colored brightening of the central visual fields that recurred intermittently. Among the responsive subjects, one (‘h5’) reported a gray-tone, visual pattern (curtain-like) that seemed to contract/expand, while another (‘h10’) reported the appearance of static horizontal line patterns. One individual (‘h9’) reported colored (green) phosphene perceived as line patterns. The visual sensations appeared diffusely over the entire visual field without the presence of any retinotopical arrangement. The phosphene perceptions, as later described by the subjects, were transient (which only lasted about a second) and occurred in a periodic fashion that were similar to the stimulation intervals.

Other than these 11 responsive subjects, two (‘h12’ and ‘h13’) reported rather transient phosphene events that occurred only a few times in the middle of the sonication experiment (categorized as ‘partially-responsive’). The other six subjects (‘h14’–‘h19’) did not report any sensations (categorized as ‘non-responsive’).

### FUS stimulation shows individual as well as group fMRI responses

**Figure 2:**
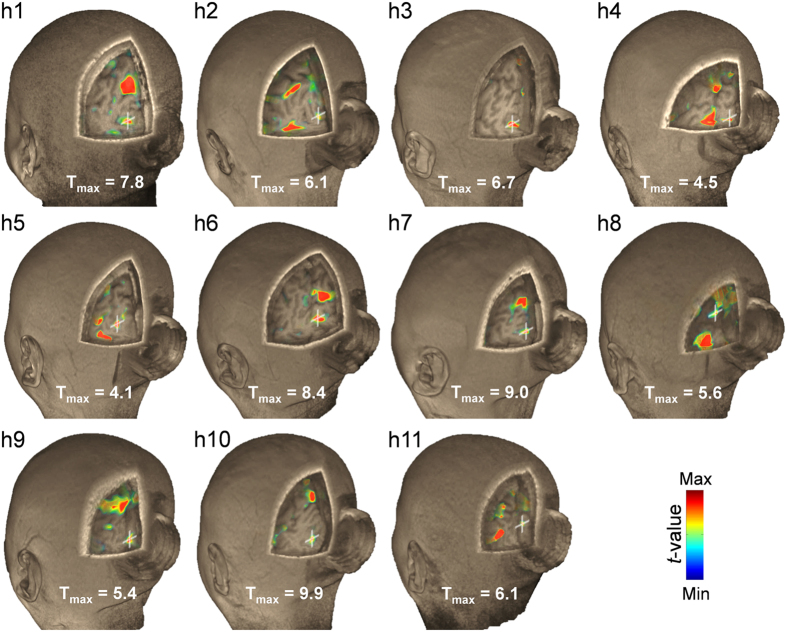
Individual fMRI results around the sonicated visual area. Pseudo-colored visualization showing the T values (*P* < 0.005, uncorrected) associated with the FUS stimulation is overlaid on a 3D-rendered high-resolution MR image volume from the responsive subjects (*N* = 11). The maximum T value is shown for each individual (T_max_) while T_min_ = 2.65. A white crosshair shows the location of the sonication target. The hollow cylindrical shape behind the head is the contour of the acoustic coupling hydrogel.

Individual-level fMRI maps (*P* < 0.005, uncorrected, Fig. 2) from responsive subjects (*N* = 11) revealed that the area showing activation in the V1 spatially aligned/overlapped with the sonication target. Furthermore, distinct activation in the V1 area was not evident from the individuals who did not report consistent phosphene (*i.e.*, ‘h12’–‘h19’; *N* = 8; data not shown). During the sham FUS session, there was no activation detected across all the participants.

**Figure 3:**
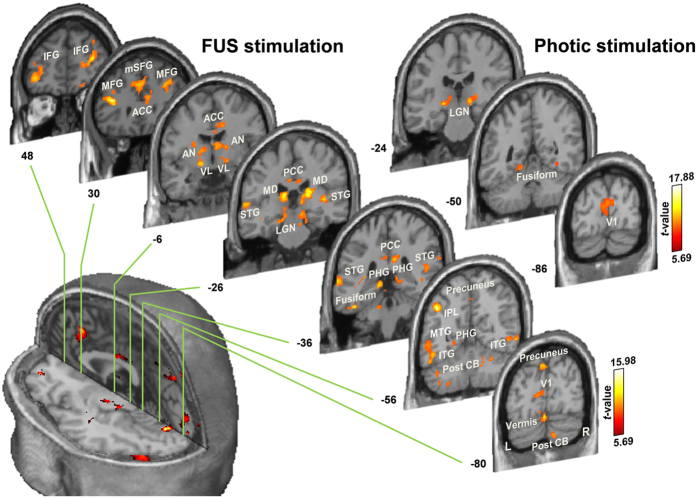
Group-level fMRI activation map elicited by sonication and photic stimulation from FUS-responsive subjects (*N* = 11) overlaid on the Montreal Neurological Institute (MNI) template MR images. Brain coordinates in the coronal direction (according to MNI coordinates) are shown next to each slice. A voxel-wise threshold of *P* < 10^−4^, with a cluster-level correction (see Methods), was used for visualization. See also Supplementary Tables S1 and S2. L, left; R, right; IFG, inferior frontal gyrus; MFG, middle frontal gyrus; mSFG, medial superior frontal gyrus; ACC, anterior cingulate cortex; PCC, posterior cingulate cortex; AN, anterior nucleus of thalamus; VL, ventral lateral nucleus of thalamus; MD, mediodorsal nucleus of thalamus; LGN, lateral geniculate nucleus of thalamus; PHG, parahippocampal gyrus; STG, superior temporal gyrus; MTG, middle temporal gyrus; ITG, inferior temporal gyrus; IPL, inferior parietal lobule; Post CB, posterior lobe of cerebellum; V1, primary visual cortex.

The group-level analysis of the fMRI data from responsive subjects (*N* = 11, thresholded at a voxel-wise threshold of *P* < 10^−4^ and cluster-level corrected *P* < 0.005 based on AlphaSim simulation^22^; see Methods) revealed that FUS activated not only the sonicated V1 but also other brain regions (Fig. 3 and Supplementary Table S1). These areas include bilateral thalamic nuclei (*i.e.*, lateral geniculate nuclei; LGN) in the primary visual pathway, along with the visual association areas, such as the precuneus, fusiform, and inferior temporal gyrus (ITG)^23^. We also found activations in the fronto-parietal areas (IFG, MFG, mSFG and IPL), cingulate cortices (ACC and PCC), and temporal lobe (STG and MTG). These are known to be neural loci that sub-serve attention networks for the regulation of cognitive processing^24^,^25^,^26^, leading us to conjecture that they were engaged during the perception of phosphene experienced by the subjects. Bilateral activations in additional thalamic areas (mediodorsal-MD and anterior/ventral lateral-AN/VL nuclei), parahippocampal gyri (PHG) and cerebellum (vermis and the posterior lobe) were also noted. Sham sonication did not elicit any activation while the photic stimulation elicited activation in the LGN and V1, along with activation in the bilateral fusiform gyrus (cluster-level corrected threshold of *P* < 0.005) (Fig. 3 and Supplementary Table S2). In the responsive group (*N* = 11, *P* < 10^−4^, cluster-level corrected threshold of *P* < 0.005), the areas showing differential activation from the ‘FUS > sham sonication’ contrast were thalamic VL, IPL, ITG (fusiform), and SFG/MFG. All of these areas also showed activations during FUS sonication (Supplementary Table S1); however, no brain activity was detected from the ‘FUS < sham sonication’ condition. Neither ‘FUS > photic stimulation’ nor ‘FUS < photic stimulation’ condition revealed differential activations (Supplementary Table S3).

The group-level analysis of the fMRI data from the subjects who did not report consistent phosphene events (*N* = 8, categorized as ‘partially-/non-responsive subjects’ above) did not reveal any activation in the brain during either the FUS stimulation or the sham sonication condition (even when using a relaxed statistical significance at a voxel-wise threshold of *P* < 10^−3^ to account for the smaller number of subjects; cluster-level corrected *P* < 0.005). However, during photic stimulation, the analysis showed activation in major visual circuitries, including the V1 and bilateral fusiform areas (V1_[x, y, z] = [2, −90, 14]_, *T*-score = 30.88, right fusiform_[x, y, z] = [24, −44, −12]_, *T*-score = 12.52, left fusiform_[x, y, z] = [−18, −52, 0]_, *T*-score = 11.82). In the partially-/non-responsive group (*N* = 8, *P* < 10^−3^, cluster-level corrected threshold of *P* < 0.005), the differential activations from ‘FUS < photic stimulation’ contrast were detected from the V1 and bilateral fusiform areas (Supplementary Table S4). There were no other differential activation detected from ‘FUS > sham sonication’, ‘FUS < sham sonication’, and ‘FUS > photic stimulation’ contrasts.

### FUS generates stimulation-specific EEG responses

Separate from the fMRI experiments, stimulation-specific EEG responses were measured from ten subjects outside of the MRI (from the occipital area, electrodes placed on either the PO7 or PO8 montage, 10–20 EEG configurations, hemisphere randomized), across the following experimental conditions—(1) resting-state, (2) FUS, (3) sham FUS, and (4) photic stimulation.

**Figure 4:**
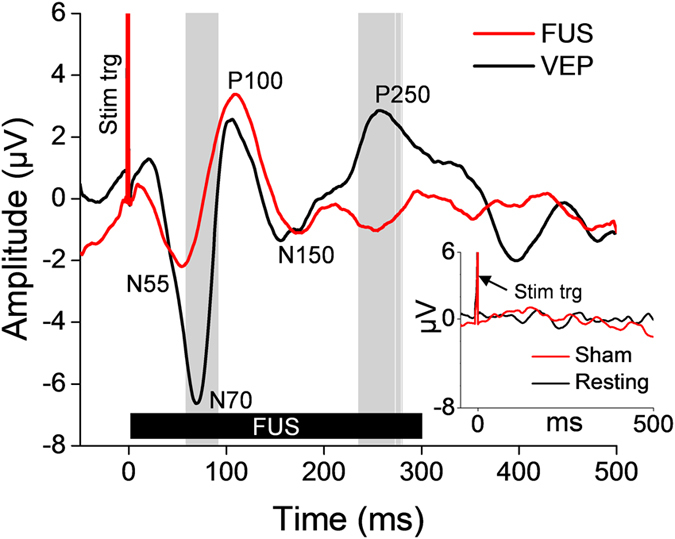
Transcranial FUS targeted to the V1 elicits the EEG evoked potentials. The grand average (*N* = 10; 50 trials per stimulation condition) EEG evoked potentials elicited by transcranial FUS to the primary visual cortex (red line) and VEP separately elicited by photic stimulation (black line). The FUS was given for a period of 300 ms (thick solid black bar labeled as ‘FUS’) while the 50 ms-long photic stimulation was given (not shown on the plot). Positive (noted with prefix P) and negative (noted with prefix N) peaks of the evoked potentials are annotated. Gray vertical bars indicate the time-segments that showed significant differences in amplitudes between the VEP and the FUS-mediated evoked potentials (*P* < 10^−3^). A spike of stimulation triggering signal is labeled as ‘Stim trig.’ The inset shows the EEG signals measured at the same electrode site from the resting-state (*i.e.*, no stimulation, black line) and the sham FUS conditions (red line).

All ten subjects who participated in the EEG recording reported phosphene perception (similar descriptors as used in the fMRI sessions, *i.e.*, diffuse brightening of the visual field, were reported). Grand average of EEG responses (*N* = 10; Fig. 4) showed that photic stimulation generated the visual evoked potentials (VEP) with distinctive components named according to their polarities (N, negative; P, positive) and latencies (in ms), *i.e.*, N70, P100, N150, and P250. The FUS sonication condition, on the other hand, elicited a negative EEG peak with around 55 ms latency after the onset of sonication (*i.e.*, N55) and a positive peak appearing at 100 ms latency (*i.e.*, P100). We found that the stimulation-related EEG responses occurred prior to the completion of the 300-ms long sonication, which indicates that shorter sonication duration may be used to elicit stimulation. The sham FUS condition did not show any distinctive peaks and was indistinguishable from that of the resting condition (Fig. 4, inset). These data show that the administration of FUS successfully stimulated the V1 and resulted in cortical evoked EEG potentials that bear similarities with those from photic stimulation.

### Acoustic simulations of transcranial FUS across the individuals

**Figure 5:**
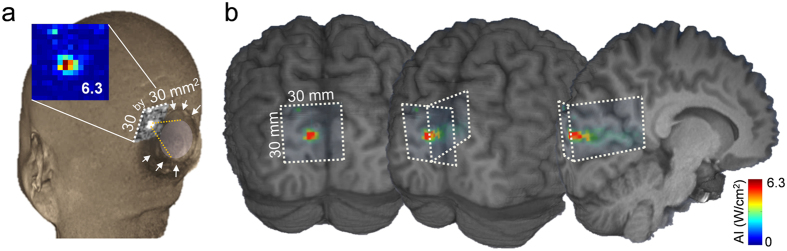
Example of simulated acoustic intensity profiles overlaid on the region of sonicated visual areas (from subject ‘h1’). (**a**) The acoustic intensity profile (30 by 30 mm^2^) of the sonication focus overlaid on a 3D rendering of the volumetric head MRI data. The shape of the acoustic coupling hydrogel is visible (surrounded by white arrows) while the sonication focus was formed in the targeted visual area (inset, colored acoustic intensity map with maximum I_sppa_ value within the simulated region-of-interest; in W/cm^2^). See also Supplementary Fig. S1. (**b**) The acoustic intensity profile projected on 3D-rendered brain sections from the same individual (from left to right, coronal section, rotated to show the mid-sagittal region of the brain). The white dotted lines indicate the boundaries of the simulated ROIs.

To assess the spatial accuracy of sonication and to estimate the level of acoustic energy experienced by each subject, we retrospectively simulated the acoustic wave propagation through the individual skull anatomy (see Methods). The acoustic intensity and mechanical index (MI) at the intended target (AI_@target,_ MI_@target_) and the maximum values within the surroundings (AI_max@ROI,_ MI_max@ROI_) were tabulated in Table 1. The spatial deviation of the FUS focus from the intended target, with the measured skull thickness along the sonication path and each subject’s responsiveness, were also shown. The simulated acoustic intensity profile (Fig. 5, pseudo-colored and overlaid on the anatomical MR images) from a subject who experienced the phosphene (‘h1’) indicates that the acoustic focus was successfully projected to the intended stimulatory area located in the calcarine fissure (see Supplementary Fig. S1 for similar data obtained from other subjects). The longitudinal profile of the simulated acoustic intensity in the direction of the beam path (Fig. 5b) also shows that most of the acoustic energy was delivered to the targeted V1 area.

The estimated I_sppa_ at the intended target ranged from 0.7 to 6.6 W/cm^2^ (average 3.0 ± 1.7 W/cm^2^; mean ± s.d., *N = *19), which was approximately 18% of the incident I_sppa_ of 16.6 W/cm^2^, suggesting that approximately 82% of the incident intensity, on average, was attenuated during the transcranial application of the FUS. The spatial deviation of the *in situ* acoustic focus from the designated target (center of the simulated region-of-interest; ROI) ranged from 0 to 16.1 mm among all subjects (average 3.3 ± 3.8 mm; mean ± s.d., *N = *19). The acoustic intensity values and the spatial error of the sonication were indistinguishable between the responsive and the partially-/non-responsive groups (*t*-test, two-tailed, *P* = 0.75 and *P* = 0.18, respectively).

### Pre-/post-sonication neurological examinations and follow-up radiological assessment

All participants underwent clinical neurological examination within one hour before and after the administration of FUS. Neurological examination conducted by three physicians (who were blinded to the nature of study) did not reveal any abnormal findings across all subjects. One female subject (‘h14’) reported a transient headache only during the fMRI session of the sham FUS condition, which was quickly resolved after the imaging data acquisition for the session. The follow-up anatomical MRI readings, conducted at three different time periods after the sonication (acute, 0.0 ± 0.0 days, *N* = 6; 2 weeks, 12.7 ± 3.2 days, *N* = 9; 4 weeks, 23.5 ± 2.4 days, *N* = 4; mean ± s.d.) did not identify any radiological abnormalities. Based on the follow-up interviews (occurred 2 months after the sonication, 60.7 ± 3.5 days; mean ± s.d., *N* = 19), there were no changes in mental or physical status or discomfort associated with the procedure.

## Discussion

Image-guided, transcranial application of FUS to the V1, along with simultaneous acquisition of fMRI, revealed strong evidence in humans that FUS activates the sonicated brain area and concurrently elicits the associated efferent sensory perception in the form of phosphene. Successful stimulation of the V1 was also supported by the presence of cortical evoked EEG potentials having similarities with the classical VEP generated by photic stimulation. The stimulatory phenomena were transient and reversible without causing any discomfort or adverse effect across the study participants.

We found that pulsed application of the FUS, using similar parameters as those from our previous studies^10^,^14^,^17^ (*i.e.*, TBD of 1 ms, PRF of 500 Hz, sonication duration of 300 ms), elicited the phosphene in more than half of the subjects (*N* = 11 responsive, *N* = 2 partially-responsive) during the fMRI and in all subjects during the EEG measurement sessions (*N* = 10). The sensation was described mostly as non-colored, non-patterned, amorphous phosphene, which occurred diffusely over the entire visual field without retinotopical arrangements. From three of the responsive subjects (‘h5’, ‘h9’, ‘h10’), patterned visual responses (‘vibrating’ or ‘line’) or colored-phosphene were reported. These types of phosphene sensations bear similarities to those reported in previous studies of TMS stimulation of the V1^27^,^28^,^29^, whereby the perception of weak lights having a pale white/gray or sometimes an ‘unsaturated color’ was reported, sometimes even accompanying the presence of textured visual patterns^30^,^31^. Although retinotopic or topographic localization in the visual responses was not seen in the present study, further systemic examination of the localized stimulation of sub-regions in the V1 would be advantageous to reveal the spatial propagation of FUS stimulation at the cortical surface as well as in depth with column organizations.

The concurrent acquisition of BOLD-contrast based fMRI revealed that the site of activation in the V1 area was spatially aligned with the sonication target (Fig. 2, evident from the individual activation maps). The subject’s perception of the phosphene, supported by the electrophysiological data, indicates that the majority of the effects may indeed stem from the actual activation from the visual area that was sonicated. This observation is in good agreement with previous functional imaging (including fMRI and PET) investigations in small animals, whereby the localized neuromodulatory effects took place at the site of the sonication focus^13^,^18^,^19^. However, acoustic pressure-mediated mechano-vascular coupling and its manifestation in BOLD signal^32^,^33^ cannot be completely ruled out as a contributing factor. Further functional mapping investigations in humans using different image-modalities, such as PET (which examines the degree of glucose metabolic activity of the brain), would help revealing the stimulatory effects of the FUS in the brain. Besides the sonicated V1 area, neural substrates in the visual pathway (such as the LGN) and visual association areas (such as precuneus, fusiform, and ITG) were activated^23^,^34^,^35^, thus sharing the neural substrates that were activated during the photic stimulation. In contrast, sham sonication did not activate any of these areas (neither from the responsive nor the partially-/non-responsive group of subjects), suggesting that neuromodulatory effects only occur as a result of sonication.

Our fMRI data suggest that the sonication not only activated the visual circuits, but also activated other remote areas of the brain (Figs 2 and 3 and Supplementary Table S1). Although definite causes for the involvement of these brain areas are difficult to ascertain, a few conjectures can be made. For example, the activations in the fronto-parietal (including IFG, MFG, mSFG, and precuneus/IPL) and temporal areas (*i.e.*, STG and MTG), together with the ACC and PCC, which all sub-serve the attention networks for cognitive processing^24^,^25^,^26^, may indicate potential engagement of attentional processes during the perception of phosphene in response to sonication^27^. In this context, the cerebellum (including vermis), departing from its traditional role in motor coordination/planning, might have been activated for the perception of phosphene due to its role in attentional processes^36^ and motion discrimination^37^. We also found that the PHG and thalamic AN/MD were bilaterally activated by the FUS stimulation, whereby these brain areas are known to participate in visual information processing, such as memory/navigation^38^ and recognition^39^,^40^. Specifically, the thalamic MD is an important substrate for the attention/intention circuits^41^ as well as executive functions^42^ as a part of cortico-striato-thalamo-cortical connections^43^. These data implicate that FUS stimulation increased neural activity from the targeted V1 area and from the network of regions involved in visual and higher-order cognitive processes, which is also supported by existing literature on TMS-induced phosphene perception^27^,^28^,^29^. Interestingly, the activations in some of these remote brain regions, *e.g.*, frontal (MFG or mSFG), temporal (MTG), and cingulate gyri (PCC), including the thalamic area (*i.e.*, VL), have also been reported from TMS stimulation of the V1 in humans^44^. This finding also indicates that the common neural substrates are activated even when different stimulation modalities are employed.

Direct comparisons between different experimental conditions (*i.e.,* FUS *versus* photic stimulation and FUS *versus* sham sonication) showed that FUS and photic stimulation resulted in similar activation patterns among responsive individuals. On the other hand, the FUS condition, when compared to the sham condition, showed a higher level of activation from thalamic areas, fusiform, and frontal gyri (Supplementary Table S3). These areas were identified as being activated in the FUS condition only. On the other hand, the partially-/non-responsive group showed greater activation in the V1 and bilateral fusiform areas during photic stimulation when compared to the FUS condition, suggesting that V1 is less likely to be activated among the individuals who did not experience phosphene.

In addition to the fMRI observation from the sonicated brain, EEG measured during the acoustic stimulation showed a distinctive positive evoked potential peak at a latency of 100 ms after the onset of sonication (Fig. 4), bearing striking similarities with the P100 component detected during the photic stimulation (associated with activation of the extrastriate cortex^45^). The FUS also elicited the N55 component, which occurred a little earlier than the N70, believed to be derived from V1 activation during photic stimulation^45^. This may suggest that the observed N55 peak stems from the neural activity at the sonicated V1 area. Differences may exist in terms of the temporal sequence of neural activation, compared to the direct sensory stimulations. The N150/P250 components detected during the photic stimulation, reported to be related to the activation of occipito-parietal areas away from the V1^45^,^46^, were not seen during acoustic stimulation. Based on these observations, along with fMRI data, we conclude that the FUS successfully stimulated the targeted visual area.

Our findings suggest that FUS can stimulate the human V1, resulting in the perception of phosphene and associated evoked potentials, while also showing a network of activated brain regions typically involved in visual and higher-order cognitive processes. The current data, therefore, does not support the spatially-selective activation of the sonicated V1 area, mostly due to the concomitant activations that were detected across the brain. Unlike functional imaging studies on anesthetized animals that showed spatially-selective activation at the sonicated region^13^,^19^, the cognitive processes from awake humans would prevent proper isolation of the brain region that was stimulated primarily by the FUS. Stimulation of unilateral somatosensory/somatomotor areas with a specific somatotopic arrangement (along with simultaneous fMRI mapping) or retinotopic creation of visual phenomena by sonicating a specific sub-region of the visual system will corroborate the spatial-selectivity of the method. Demonstration of selective stimulation of the deep brain area that is beyond the reach of the TMS technique (for example, subcortical thalamic nuclei) and the safety of the method awaits further investigations.

Based on the numerical simulation of acoustic intensity distribution around the sonicated V1 area, we found that the use of 270 kHz fundamental frequency, lower than that used in our previous small animal studies^10^,^13^, provided adequate acoustic transmission of the sonication through the skull. In most cases, the estimated location of the FUS focus was closely aligned with the target-of-interest (Table 1). It is interesting, however, to note that a few individuals (‘h12’–‘h16’, including partially responsive subjects, *N* = 5) did not report robust and repeated perception of the phosphene even though spatially-accurate sonication was given at an acoustic intensity level (*i.e.*, 2.7–6.6 W/cm^2^ I_sppa_) that was comparable to that given to the responsive individuals. We conjecture that this seemingly inconsistent perception of phosphene events may be attributed to the existence of individual variabilities/differences in the threshold acoustic intensity level for successful FUS stimulation, whereby a similar tendency has also been observed in previous studies involving both humans^17^ and animals^11^,^14^,^47^. The variability of the threshold in eliciting neural responses was also seen in TMS-mediated phosphene in humans (see Deblieck *et al.*^48^). To explain the large individual variability in responses that was observed, a measure of subject responses to different FUS intensities/parameters would be desirable. Alternatively, ‘active’ control conditions (for example, sonicating different locations within the V1) would have been valuable and constitutes a subject for future investigation. While the ultrasound-mediated alteration of cellular transmembrane capacitances/potentials^49^ or involvement of the glial systems^50^ has been hypothesized to be associated with the stimulatory phenomena, unveiling the underlying mechanism of acoustic neural stimulation would provide the key to explain these variabilities, or even perhaps reduce them. Further study on temporal sequence of cell-level neural activation with respect to the acoustic stimulus may also help to elucidate the mechanism.

Among a subset of subjects who did not report any visual sensations (*i.e.*, ‘h17’–‘h19’, *N* = 3), the simulation revealed a rather large spatial error in sonication (ranging 7.2–16.1 mm from the targeted V1, Table 1). We conjecture that the differences in local skull shapes, such as osseous grooves often seen in the inner wall of the occipital-cerebellar junction of the skull, which may be present in the sonication path, might have resulted in an alteration of the propagation of the acoustic waves through the skull. In this context, guidance of the sonication path using on-site acoustic simulation with acoustic aberration corrections^51^ would further improve the spatial accuracy of transcranial FUS.

Through the neurological and neuroimaging examinations, the sonication parameters used in the present study appear to be safe, with a maximum estimated acoustic intensity of 11.6 W/cm^2^ I_sppa_ (experienced in ‘h5’). The corresponding MI was 1.2, which is much lower than the US Food and Drug Administration (FDA) safety guideline limit of 1.9 for soft tissue sonication^52^. The estimated I_spta_ at the target, averaged across the subjects, was on the order of 1.5 ± 0.9 W/cm^2^ (based on the 50% duty cycle of 3.0 ± 1.7 W/cm^2^ I_sppa_; Table 1). This was lower than 3 W/cm^2^ I_spta_, which is in compliance with the international electrotechnical commission (IEC) 60601 part 2 standard for therapeutic equipment^52^. These results, along with the accumulating safety records from previous studies^10^,^13^,^14^,^15^,^16^,^17^, cast a promising feasibility for the safe administration of neuromodulatory FUS in humans. However, caution is needed to avoid the use of sonication at extremely high repetitions (>500 times and more) in short intervals (*e.g.*, given every second) since this may cause microhemorrhages in the brain parenchyma, based on our recent observation from ovine experiments^14^.

Our results suggest that FUS can serve as a novel non-invasive stimulation technique in conscious humans. The emerging capabilities of FUS as a non-invasive stimulation modality of region-specific areas in the human brain may lead to many applications, for example, assessment of the regional brain functions and their functional connectivity to different parts of the brain. Along with its ability to modulate levels of neurotransmitters^53^,^54^, FUS may also be able to provide a new mode of neurotherapeutics for remedying various types of pathological conditions associated with aberrant neurotransmissions. Optimization of acoustic parameters for effective and safe sonication as well as further systemic investigations for revealing the detailed mechanism of acoustic neuromodulation would help to realize these potential applications.

## Methods

### Participants and study overview

The study was conducted in accordance with the ethical guidelines set forth by the Institutional Review Boards (IRBs) of both the Catholic University of Korea (FUS planning *via* functional neuroimaging and neuro/radiological assessment of study participants) and the Korea University (FUS-mediated brain stimulation and its fMRI/EEG characterization). All experimental protocols were approved by the IRBs of both universities. A total of 19 healthy volunteers participated (female = 5; age 20–45, average 26.1 ± 5.4) and informed consent was obtained from all subjects prior to the study procedures. The experiment consisted of two separate procedures: (1) MRI/CT imaging and (2) transcranial FUS sonication, which occurred on different days (gap between the procedures 14.8 ± 22.4 days; mean ± s.d., *N* = 17; two participants had existing MRI/CT data). In the first procedure, the study participants underwent anatomical/functional MRI to map their visual areas for the sonication planning (See Supplementary Methods). Cranial CT was acquired to screen for the presence of intracranial calcification and to provide information on skull geometry. In the second procedure, the subject underwent an MR-guided FUS session, whereby simultaneous acquisition of BOLD-sensitive fMRI was used to detect the brain areas that were activated by the FUS stimulation. As a separate experimental session, we also measured the EEG evoked potentials associated with FUS and photic stimulation from a few participants (*N* = 10, female = 1, age 20–45, average 26.7 ± 7.1) outside of the MRI.

### Sonication planning and image-guided FUS to the primary visual cortex

Functional/anatomical MRI and CT data were mutually co-registered using the same technique as described in our previous work^17^. Prior to the actual sonication session, the co-registered MRI/CT data were used to plan the sonication path to the target in the V1 while maintaining the entry angle as perpendicular as possible to the skull surface (Fig. 1c).

On the day of the sonication experiment, the subject underwent neurological examination by certified neurologists. The FUS sonication was then performed under the guidance of a 3-Tesla MR scanner (MAGNETOM TrioTim, Siemens; 12-channel head coil). The subject’s anatomical features with respect to the inion (*i.e.*, external occipital protuberance) were used to initially position the head with respect to the transducer, and subsequent T1–weighted MR imaging (GRAPPA sequence, acceleration factor = 2, TR/TE = 1,900/2.52 ms, flip angle = 9°, slice thickness = 1 mm, FOV = 25.6 × 25.6 cm^2^, image matrix = 256 × 256, voxel size = 1 × 1 × 1 mm^3^) was performed to visualize the profile of the FUS transducer (represented in Figs 1d, 2 and 5a; visible as a negative contour surrounded by the water-rich hydrogel^21^) and the spatial distortion in the image was minimal. The use of the high-resolution (1 mm^3^ isotropic voxel dimension), T1-weighted anatomical images using a short echo time (2.52 ms) effectively revealed the 3D geometry of the transducer for guidance. The identification of the transducer allowed for the estimation of spatial coordinates of the sonication focus as well as beam orientation with respect to the subject’s neuroanatomy. If the location of the acoustic focus and orientation of the sonication path grossly deviated from the intended plan, the head was carefully repositioned and imaging was repeated until the sonication focus fell approximately within 1 cm from the target.

Event-related fMRI was conducted during three different types of experimental conditions to capture the neural activity associated with each condition—(1) FUS, (2) sham FUS, and (3) photic stimulation (without FUS). These conditions were randomized and balanced across the participants. Being synchronized with the MR scanner operation, the 300-ms long stimulation (either FUS sonication or photic stimulation) was given 50 times every 13 s. The ambient light was turned off. The FUS session was conducted with the subject’s eyes closed, whereas the photic stimulation session was performed with the subject’s eyes open. 25 Hz flickering checkerboard presentation was used as the photic stimulation (so that at least eight flickers can be shown during 300 ms) *via* MR-compatible goggles (NordicNeurolab, Bergen, Norway). The sham FUS condition, administered under the same FUS procedures but without actual sonication, was not discernable to the participants. The fMRI data were acquired covering the entire brain volume using a gradient-echo echo planar imaging (EPI) sequence (TR/TE = 2,200/30 ms, flip angle = 90°, slice thickness = 3 mm, FOV = 22.0 × 22.0 cm^2^, image matrix = 64 × 64, voxel size = 3.44 × 3.44 × 3.00 mm^3^, 40 slices).

### fMRI data processing

The acquired fMRI data was processed using the SPM8 software, whereby the stimulation-related neuronal activity was estimated by the general linear model (GLM) after slice-timing and motion corrections. The Gaussian smoothing kernel with [6 × 6 × 6] FWHM was applied in volumetric images. The voxel-wise statistic parametric map, with respect to the stimulation-specific canonical hemodynamic response function (HRF), was obtained. For the identification of the individual-level activation, the T1 anatomical images were co-registered to the EPI image space. For the derivation of the group-level activation map, the data were further divided into two groups based on the subjective reporting of any visual phenomena during the FUS session (Table 1, responsive group, *N* = 11; and partially-/non-responsive groups, *N* = 8) and were subjected to random effect analysis (RFX)^55^ across the different stimulation conditions (*i.e.*, FUS, sham FUS, and photic stimulation). Subsequently, we used a threshold level *P* < 10^−4^, with corrections based on cluster extent thresholds^56^ to reject false positives using the REST AlphaSim toolbox^22^ (www.restfmri.net; toolkit v1.8; 1000 Monte Carlo simulations were used).

### Electrophysiological representation of the FUS stimulation

Separate from the fMRI experiments, sonication-specific EEG responses from the visual areas were measured from ten subjects outside of the MRI (‘h1’–‘h3’, ‘h5’, ‘h6’, ‘h8’–‘h11’, and ‘h17’). The EEG session was conducted using an identical sonication setup as the FUS-MRI session, but without the inclusion of the RF filters. Before and after each EEG-FUS session, another set of neurological examination was given. The subject lay supine and the head was positioned over the sonication apparatus, as guided by the subject’s anatomical features (*e.g.*, the inion). All subjects were asked to close both eyes during the following experimental conditions—(1) resting-state without any external stimuli, (2) FUS stimulation of the visual cortex (using the parameters used in the fMRI session; 300-ms long sonication), (3) sham FUS condition (mimicking the sonication environment without providing actual sonication), and (4) photic stimulation (50 ms-long white LED lighting with a diffusive filter in front of the eyes). These conditions were randomized and balanced across the participants. The stimulation (FUS, sham FUS, or photic) was given 50 times every 2.5 s and the corresponding EEG responses were measured in a time-locked fashion (*i.e.*, data was not acquired continuously) from time segments around the stimulations (*i.e.*, −50 ms to 500 ms with respect to the simulation onset) with an inter acquisition interval of 2.5 s. Resting-state EEG acquisition was performed in the same fashion without any external stimuli. The EEG data were acquired from adhesive electrodes (Covidien, Mansfield, MA) placed on either the PO7 or PO8 montage (hemispheric side randomized for detecting the VEP; according to the international 10–20 system) using data acquisition hardware (ML408; ADInstruments, CO) and software (PowerLab, PL3504/P; ADInstruments, CO). A reference electrode was applied to the ipsilateral (right) mastoid and a ground electrode was placed on the Fpz. The data were online filtered with a 60 Hz notch filter and a bandpass filter (0.3–200 Hz) prior to undergoing subsequent off-line analysis. Baseline correction, including linear detrending, was not conducted. For artifact removal, the EEG time series having data points out of the data acquisition/detection range (−100 μV to 100 μV) were manually excluded from further analysis.

The grand mean trace was computed by averaging EEG evoked potentials (*N* = 10; 50 trials per stimulation condition) elicited during each experimental condition. Statistical analyses were performed on the time-series of EEG responses using Matlab (Mathworks, Natick, MA), comparing the mean EEG amplitude at each time point (1-ms stepwise) between the FUS and photic stimulation conditions. In order to control for multiple comparison problems encountered in analyses of complex EEG data sets, we analyzed the EEG data based on the nonparametric permutation technique^57^. Permutation tests were performed whereby the *p*-value was computed from the proportion of 1,000 permuted (randomized) surrogates having a test statistic larger than the *t*-value from conventional paired *t*-test (two-tailed)^57^. *P* < 10^−3^ was considered as significant differences between the VEP and the FUS-mediated evoked potentials. We measured the time latency between the onset of stimulation to the peaks from the grand average evoked potentials (*N* = 10) using LabChart 7 (ADInstruments, CO).

### Subject-specific acoustic simulations and estimation of thermal effects from the sonication

Direct *in vivo* visualization of the acoustic pressure profile (for example, utilization of the MR acoustic radiation force impulse [ARFI] technique^58^) is difficult at the level of acoustic intensity used in this study, and the heating of local brain tissue (for MR thermometry^59^,^60^ detection of temperature changes) is not applicable to healthy individuals. Therefore, we estimated the location and acoustic intensity of the FUS focus inside of the brain using acoustic simulation software (Wave3000; Cyberlogic, New York, NY) as described previously^17^. The variables of the material properties were used as the values provided by the software (*ρ* = 1850 kg/m^3^, *λ* = 9306 MPa, *μ* = 3127 MPa, *η* = 40 Pa·s, *ϕ* = 0.1 Pa·s for skull; *ρ* = 1000 kg/m^3^, *λ* = 2241 MPa, *μ* = 0 MPa, *η* = 0.001 Pa·s, *ϕ* = 1.0 × 10^−7^ Pa·s for water; where *ρ* = density, *λ* = Lamé's first parameter related to the bulk modulus and shear modulus, *μ* = Lamé’s second parameter or the rigidity modulus, *η* = the shear or first viscosity, *ϕ* = the bulk or second viscosity). Conjointly, the acoustic attenuation by the neural tissue on the specific sonication path was included in the estimation using the attenuation factor of the brain tissue (*i.e.,* 0.0175 Np/cm)^61^ and the intracranial distance. For the simulation, the location and orientation of the transducer with respect to the anatomy was determined from the volumetric MR data (Fig. 1d). Individual skull CT data co-registered to the MRI space were used to estimate the acoustic intensity distributions in a 30 × 30 mm^2^ square ROI around the center of the intended target perpendicular to the sonication path. In one subject (‘h1’), simulation was also conducted to visualize the estimated acoustic intensity profile in a longitudinal plane along the centroid of the sonication path, covering a 30 × 44 mm^2^ rectangular ROI around the intended target. Another set of simulations, but excluding the skull anatomy, was conducted as a control condition to assess the degree of acoustic intensity transmission and spatial deviation of the acoustic focus from the intended target. At each acoustic intensity level, a corresponding MI, which describes the likelihood of biological effects due to cavitation in tissues^62^, was also calculated (Table 1).

The maximum level of theoretical thermal increase (ΔT) was estimated from the maximum *in situ* level of simulated acoustic intensity (*i.e.*, 11.6 W/cm^2^ I_sppa_ experienced by subject ‘h5’, see Table 1, AI_max@ROI_) using the equation ΔT = 2αIt/ρ_b_C_p_^63^ = 2 × 0.0175 cm^−1^ × 5.8 W·cm^−2^ × 0.3 s/3.811 J·cm^−3^·°C^−1^; where α = absorption coefficient^61^, I = attenuated I_spta_ at the FUS focal area, t = sonication duration, ρ_b_ = density of brain tissue^64^, and C_p_ = specific heat of the brain tissue^64^. The estimated ΔT was 0.016 °C, which is far below the temperature threshold that can cause any discernible thermal effects on brain tissues^65^.

## Additional Information

**How to cite this article**: Lee, W. *et al.* Transcranial focused ultrasound stimulation of human primary visual cortex. *Sci. Rep.* **6**, 34026; doi: 10.1038/srep34026 (2016).

## Supplementary Material

Supplementary Information

## Figures and Tables

**Table 1:** The individual-specific acoustic simulation results of the FUS stimulation to the V1.

ID	Gender	AI_@target_ I_sppa_ (W/cm^2^)	AI_max@ROI_ I_sppa_ (W/cm^2^)	MI_@target_	MI_max@ROI_	Deviation from the focus (mm)	Skull thickness (mm)	Subject responsiveness
h1	M	4.8	6.3	0.8	0.9	2.0	5.7	Yes
h2	F	1.2	3.7	0.4	0.7	4.0	6.8	Yes
h3	M	5.6	7.0	0.8	0.9	2.0	2.8	Yes
h4	F	2.7	4.5	0.6	0.7	2.0	5.6	Yes
h5	M	4.0	11.6	0.7	1.2	2.0	3.8	Yes
h6	M	2.3	3.2	0.5	0.6	4.0	7.8	Yes
h7	M	2.0	7.0	0.5	0.9	2.0	6.7	Yes
h8	M	1.5	1.7	0.4	0.4	2.0	6.7	Yes
h9	M	3.7	6.3	0.7	0.9	2.0	4.7	Yes
h10	M	1.3	3.2	0.4	0.6	4.0	6.7	Yes
h11	M	2.3	2.3	0.5	0.5	0.0	5.6	Yes
h12	M	5.3	5.3	0.8	0.8	0.0	2.1	Partial
h13	M	6.6	9.1	0.9	1.1	2.0	2.3	Partial
h14	F	3.2	5.1	0.6	0.8	2.0	6.6	No
h15	M	2.7	5.5	0.6	0.8	2.0	4.8	No
h16	M	3.7	3.7	0.7	0.7	0.0	6.7	No
h17	M	0.7	4.8	0.3	0.8	16.1	7.8	No
h18	F	1.2	1.7	0.4	0.4	8.2	6.9	No
h19	F	1.7	3.7	0.4	0.7	7.2	8.7	No
	Mean	3.0	5.0	0.6	0.8	3.3	5.7	
	s.d.	1.7	2.5	0.2	0.2	3.8	1.9	

The estimated acoustic intensity, in terms of I_sppa_ and the corresponding mechanical index at the intended target location (AI_@target_, MI_@target_) and the maximum values within the surroundings (AI_max@ROI_, MI_max@ROI_), along with the estimated spatial deviations in targeting during the fMRI-FUS session. See also Supplementary Fig. S1. Each subject’s skull thickness measured along the sonication path and responsiveness to the stimulation are also shown. Note that the incident acoustic intensity was 16.6 W/cm^2^ I_sppa_. M and F denote male and female, respectively.
