# Supplementary material for: Transcranial focused ultrasound stimulation of human primary visual cortex
Source: Sci Rep. 2016 Sep 23;6:34026. doi: 10.1038/srep34026 (PMC5034307; doi:10.1038/srep34026)
Supplement: Supplementary Information [file srep34026-s1.doc]

**Supplementary Information**

Transcranial focused ultrasound stimulation of
human primary visual cortex

Wonhye Lee^2,3^, Hyun-Chul Kim^1^, Yujin Jung^2^, Yong An Chung^2^, In-Uk Song^2^, Jong-Hwan Lee^1^ and Seung-Schik Yoo^2,3,*^

^1^Department of Brain and Cognitive Engineering, Korea University, Seoul, Korea

^2^Incheon St. Mary’s Hospital, The Catholic University of Korea, Incheon, Korea

^3^Department of Radiology, Brigham and Women’s Hospital, Harvard Medical School, Boston, MA, USA

Supplementary Figure

**Supplementary Figure S1.**

Supplementary Tables

**Supplementary Table S1.** **Brain anatomical regions showing activation elicited by FUS sonication**

**Supplementary Table S2.** **Brain anatomical regions showing activation during photic stimulation**

**Supplementary Table S3. Brain anatomical regions showing differential activations across the FUS sonication, photic stimulation, and sham FUS condition in the responsive group (*N* = 11).**

**Supplementary Table S4. Brain anatomical regions showing differential activations across the FUS sonication, photic stimulation, and sham FUS conditions in the partially-/non-responsive group (*N* = 8).**

Supplementary Methods

**Multi-modal image acquisition and processing for sonication planning**

**The sonication setup**

**Characterization of the FUS acoustic field and operating parameters**

**Post-FUS session follow-up**

Supplementary References

Supplementary Figure


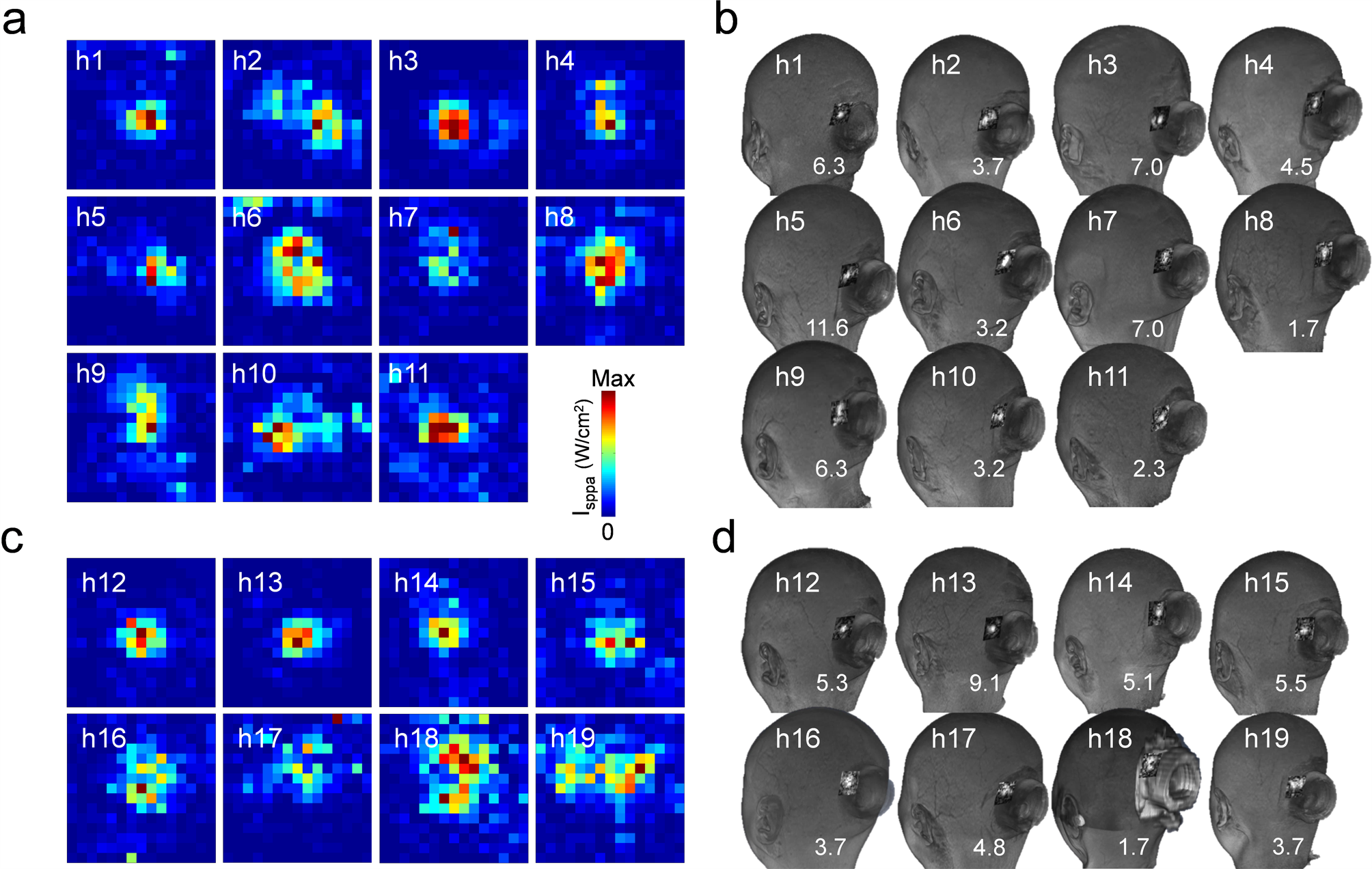


**Supplementary Figure S1.** **Simulated acoustic intensity profiles on a transversal plane (perpendicular to the sonication path, 30 × 30 mm^2^ square area) around the center of the FUS focus targeted to the primary visual cortex.** The acoustic intensity profiles projected on (**a**) the transversal plane and (**b**) a 3D rendering of the volumetric MRI data among the subjects who experienced phosphene (*N* = 11). The simulated acoustic intensity profiles on (**c**) the transversal plane and (**d**) a 3D rendering of the volumetric MRI among partially-/non-responsive subjects (*N* = 8). The number under each 3D-rendered head anatomy denotes the maximum acoustic intensity value within the region-of-interest (in I_sppa_; W/cm^2^)

Supplementary Tables

**Supplementary Table S1.** **Brain anatomical regions showing activation elicited by FUS sonication**

| **Region** | **Cluster size (voxels)** | **x** | **y** | **z** | **Peak *T* score** |
| --- | --- | --- | --- | --- | --- |
| Middle frontal gyrus, R | 945 | 28 | 56 | -14 | 13.23 |
| Inferior frontal gyrus, R |  | 40 | 38 | 4 | 10.57 |
| Middle frontal gyrus, L | 1076 | -38 | 42 | 18 | 11.65 |
| Inferior frontal gyrus, L |  | -38 | 32 | 20 | 14.68 |
| Medial superior frontal gyrus, M | 366 | 0 | 32 | 34 | 9.10 |
| Anterior cingulate cortex, M |  | 4 | 36 | 24 | 8.29 |
| Thalamus, ventral lateral/anterior nucleus, R | 1448 | 20 | -6 | -8 | 7.85 |
| Thalamus, ventral lateral/anterior nucleus, L |  | -14 | -4 | -6 | 9.58 |
| Thalamus, mediodorsal nucleus, R |  | 10 | -22 | 14 | 13.03 |
| Thalamus, mediodorsal nucleus, L |  | -12 | -26 | 18 | 11.46 |
| Thalamus, lateral geniculate nucleus, R |  | 10 | -26 | -10 | 7.82 |
| Parahippocampal gyrus, R |  | 16 | -34 | 0 | 8.19 |
| Thalamus, lateral geniculate nucleus, L | 279 | -14 | -26 | -12 | 7.71 |
| Parahippocampal gyrus, L |  | -12 | -36 | 0 | 9.91 |
| Posterior cingulate cortex, M | 349 | 8 | -34 | 26 | 9.40 |
|  |  | -8 | -40 | 28 | 9.68 |
| Superior temporal gyrus, R | 262 | 42 | -24 | 4 | 10.56 |
| Superior temporal gyrus, L | 188 | -66 | -38 | 14 | 10.31 |
|  | 105 | -44 | -20 | -6 | 8.47 |
| Inferior temporal gyrus (Fusiform), L | 455 | -44 | -36 | -22 | 12.70 |
| Middle temporal gyrus, L |  | -50 | -68 | -12 | 9.07 |
| Inferior temporal gyrus (Fusiform), R | 135 | 44 | -8 | -30 | 9.33 |
| Inferior temporal gyrus, R | 394 | 42 | -38 | -2 | 15.73 |
| Inferior parietal lobule, L | 198 | -44 | -56 | 44 | 15.70 |
| Precuneus (BA7), R/L | 291 | 8 | -74 | 52 | 7.53 |
|  |  | -2 | -80 | 46 | 9.54 |
| Primary visual cortex (BA17/18), R/L | 382 | -8 | -86 | 8 | 7.65 |
|  |  | 2 | -94 | 8 | 7.93 |
| Cerebellum, vermis, M | 425 | 0 | -80 | -16 | 10.88 |
| Cerebellum, superior posterior lobe, L | 257 | -40 | -66 | -32 | 9.39 |
| Cerebellum, inferior posterior lobe, L | 155 | -30 | -62 | -48 | 9.93 |
| Cerebellum, superior posterior lobe, R | 232 | 32 | -64 | -26 | 4.59 |
| Cerebellum, inferior posterior lobe, R | 125 | 20 | -72 | -44 | 10.39 |

Anatomical coordinates of significant clusters (thresholded at a voxel-wise threshold *P* < 10^-4^ with a cluster extent threshold of 77 voxels; corrected *P* < 0.005) of group-level activation (*N* = 11) elicited by FUS. Multiple anatomical regions were identified within a single cluster. The x, y, z coordinates are given in peak MNI space coordinates. L, M, and R denote left, medial, and right, respectively.

**Supplementary Table S2.** **Brain anatomical regions showing activation during photic stimulation**

| **Region** | **Cluster size (voxels)** | **x** | **y** | **z** | **Peak *T* score** |
| --- | --- | --- | --- | --- | --- |
| Thalamus, lateral geniculate nucleus, R | 60 | 14 | -24 | -2 | 9.93 |
| Thalamus, lateral geniculate nucleus, L | 91 | -20 | -16 | 0 | 17.88 |
| Fusiform, R | 173 | 36 | -62 | -14 | 10.26 |
| Fusiform, L | 67 | -20 | -38 | -18 | 7.05 |
| Primary visual cortex (BA17/18), R/L | 366 | 12 | -84 | 16 | 7.90 |
|  |  | -4 | -88 | 16 | 7.68 |

Anatomical coordinates of significant clusters (thresholded at a voxel-wise threshold *P* < 10^-4^ with a cluster extent threshold of 46 voxels; corrected *P* < 0.005) of group-level activation (*N* = 11) elicited by photic stimulation. The x, y, z coordinates are given in peak MNI space coordinates. L and R denote left and right, respectively.

**Supplementary Table S3. Brain anatomical regions showing differential activations across the FUS sonication, photic stimulation, and sham FUS condition in the responsive group (*N* = 11)**

| **Region** | **Cluster size (voxels)** | **x** | **y** | **z** | **Peak *T* score** |
| --- | --- | --- | --- | --- | --- |
| **FUS > Photic stimulation** | | | | | |
| None detected | - | - | - | - | - |
| **FUS < Photic stimulation** | | | | | |
| None detected | - | - | - | - | - |
| **FUS > Sham sonication** | | | | | |
| Thalamus, ventral lateral nucleus, L | 358 | -14 | -10 | 10 | 6.93 |
| Cerebellum, inferior posterior lobe, L | 174 | -40 | -50 | -46 | 6.46 |
| Inferior temporal gyrus (Fusiform), R | 92 | 44 | -14 | -28 | 6.20 |
| Superior frontal gyrus, L | 98 | -20 | 54 | 12 | 5.72 |
| Middle frontal gyrus, L |  | -28 | 58 | 16 | 5.40 |
| **FUS < Sham sonication** | | | | | |
| None detected | - | - | - | - | - |

Anatomical coordinates of significant clusters (thresholded at a voxel-wise threshold *P* < 10^-4^; corrected *P* < 0.005) of group-level differential activations across the different experimental conditions in the responsive group (*N* = 11). The x, y, z coordinates are given in peak MNI space coordinates. L and R denote left and right, respectively.

**Supplementary Table S4. Brain anatomical regions showing differential activations across the FUS sonication, photic stimulation, and sham FUS conditions in the partially-/non-responsive group (*N* = 8)**

| **Region** | **Cluster size (voxels)** | **x** | **y** | **z** | **Peak *T* score** |
| --- | --- | --- | --- | --- | --- |
| **FUS > Photic stimulation** | | | | | |
| None detected | - | - | - | - | - |
| **FUS < Photic stimulation** | | | | | |
| Fusiform, R | 138 | 18 | -58 | -8 | 5.696 |
| Fusiform, L | 122 | -26 | -54 | -14 | 5.539 |
| Primary visual cortex (BA17/18), R | 110 | 12 | -80 | 14 | 5.678 |
| **FUS > Sham sonication** | | | | | |
| None detected | - | - | - | - | - |
| **FUS < Sham sonication** | | | | | |
| None detected | - | - | - | - | - |

Anatomical coordinates of significant clusters (thresholded at a voxel-wise threshold *P* < 10^-3^; corrected *P* < 0.005) of group-level differential activations across the different experimental conditions in the partially-/non-responsive group (*N* = 8). The x, y, z coordinates are given in peak MNI space coordinates. L and R denote left and right, respectively.

Supplementary Methods

**Multi-modal image acquisition and processing for sonication planning**

A 3-Tesla clinical MR scanner (MAGNETOM Skyra, Siemens) was used to obtain anatomical and functional information from the brain using a 4-channel head coil. First, anatomical T1-weighted images (3D GRAPPA sequence, acceleration factor = 2, repetition time (TR) = 1,900 ms, echo time (TE) = 2.46 ms, flip angle = 9º, slice thickness = 0.94 mm, field-of-view (FOV) = 24 × 24 cm^2^, image matrix = 256 × 256, voxel size = 0.94 × 0.94 × 0.94 mm^3^, 192 slices) were acquired in the sagittal orientation covering the entire telencephalic areas of the head.

To identify the individual-specific location of the visual areas of the brain, fMRI was performed using a gradient-echo echo-planar-imaging (EPI) sequence (TR/TE = 2,500/30 ms, flip angle = 90º, slice thickness = 4 mm, FOV = 24 × 24 cm^2^, image matrix = 96 × 96, voxel size = 2.5 × 2.5 × 4 mm^3^, 40 slices). The orientation of the image slice was set parallel to the imaginary plane connecting the anterior commissure (AC) and the posterior commissure (PC). Visual areas were activated by presenting photic stimulation (25 s-long; checkerboard pattern flickering at 8 Hz and at a viewing angle of ~11°) interleaved by a fixation condition (25 s-long; crosshair in the middle of the screen) on an MR-compatible screen (ESys fMRI, In Vivo, Gainesville, FL). The three blocks of the stimulation were interleaved by four fixation conditions of equal duration. A dummy scan of 7.5 s was included at the beginning of the imaging session to allow for T1 signal equilibration and was excluded from further data processing.

The fMRI data was processed using the SPM8 software package (Wellcome Department of Imaging Neuroscience, University College London, London, UK; www.fil.ion.ucl.ac.uk/spm), whereby the task-related neuronal activity was estimated by a general linear model (GLM) after slice-timing and motion corrections. With respect to the stimulation-specific canonical hemodynamic response function (HRF), the voxel-wise statistical parametric map was obtained, and a threshold *P* < 0.005 (uncorrected) was applied to the map for the visualization of the activation. The local maximum of the activated area around the calcarine fissure was set as the sonication target, and the coordinates were recorded for later FUS sessions. The anatomical information of the skull was obtained using a clinical CT scanner (Aquilion ONE, Toshiba, Japan), which imaged most of the participant’s head (axial orientation, slice thickness = 0.5 mm, FOV = 24 × 24 cm^2^, image matrix = 512 × 512, voxel size = 0.47 × 0.47 × 0.50 mm^3^, number of slices varied according to the subject’s head size).

**The sonication setup**

A schematic of the FUS sonication setup is shown in **Figure 1a**. The entire sonication apparatus was made with non-ferromagnetic materials and is compatible in an MR environment. A ceramic piezoelectric FUS transducer (Channel Industries, Santa Barbara, CA), having the shape of a segmented-sphere (an outer diameter of 3 cm; focal length of 3 cm), was housed in an air-backed, custom-made plastic enclosure. The transducer was actuated at a fundamental frequency of 270 kHz and was mounted to the pillow-shaped plastic enclosure, whereby compressible acoustic hydrogel (polyvinyl alcohol; PVA)^1^ was applied to the front of the transducer to couple the acoustic path between the transducer surface and the scalp. The PVA hydrogel, having a (compressed) thickness of ~1 cm, allowed for approximately 2 cm depth penetration of the acoustic focus to the visual cortex, as the focal length of the FUS transducer is 3 cm. The FUS transducer assembly was placed inside of the MR head coil. After carefully combing away the subject’s hair from the sonication entry site, ultrasound hydrogel (Aquasonic, Parker Laboratories, Fairfield, NJ) was applied to the scalp. The subject lay supine and the head was positioned over the sonication apparatus (**Fig. 1a**) with soft cushions around the head and neck to discourage movement during the sonication and fMRI. The FUS transducer, when powered, did not generate audible sounds or tangible peripheral sensations on the scalp.

The input signal to the transducer was generated by two signal generators (33210A; Agilent technologies, Inc., Santa Clara, CA) and was subsequently amplified by a class-A power amplifier (Electronics and Innovations, Rochester, NY) with an impedance matching circuit^2^. Then, the signal was low-pass filtered (cutoff frequency < 1.9 MHz, BLP-1.9+, MiniCircuits, Brooklyn, NY) to deliver the electrical power to the transducer through a wall panel for compatibility with the MR environment. Prior to the experiment, we checked for the absence of any undesirable image artifacts that could be introduced by the sonication setup (such as radio-frequency [RF] interferences in the image- as well as time-domain MR data), by imaging a water-filled cylindrical phantom and subsequently examining pixel-by-pixel intensity variations along the time-domain. We also confirmed that the quality of the EPI images (of the brain) was not affected by the FUS transducer through a test EPI acquisition (TR/TE = 2,200/30 ms, flip angle = 90º, slice thickness = 3 mm, FOV = 22.0 × 22.0 cm^2^, image matrix = 64 × 64, voxel size = 3.44 × 3.44 × 3.00 mm^3^, 40 slices) for each subject prior to the fMRI-FUS session.

**Characterization of the FUS acoustic field and operating parameters**

Detailed methods for transducer characterization (for example, mapping of the acoustic pressure field) and the derivation of acoustic intensities at the focus with respect to the input voltage of the driving electrical signal are described elsewhere^2^. The spatial profile of the acoustic intensity at the focus was measured in the transverse plane perpendicular to the incident sonication beam path (31 × 31 mm^2^ square area, 1 mm step) as well as parallel to the beam (measured with a 20 mm gap from the exit plane of the transducer, 31 × 61 mm^2^ rectangular area, 1 mm step) using a needle hydrophone (HNC-200, Onda, Sunnyvale, CA). The size of the focus was 3 mm in diameter and 17 mm in length, as defined by the FWHM of the acoustic intensity map (**Fig. 1b**, upper panel).

For the acoustic stimulation, we adapted a similar pulsing scheme that was used in the successful stimulation of the somatosensory cortex in humans^3^ and animals^4,5^, having a TBD of 1 ms with a PRF of 500 Hz (*i.e.*, duty cycle of 50%) and a sonication duration of 300 ms (illustrated in **Figure 1b**, lower panel). The incident acoustic intensity at the FUS focus, without the presence of the skull, had an I_sppa_ of 16.6 W/cm^2^, resulting in an I_spta_ of 8.3 W/cm^2^.

**Post-FUS session follow-up**

After each experimental session (fMRI or EEG sessions), the participants were asked to describe any sensations using their own words. Experimenters avoided using any words suggesting any specific sensory modality throughout the entire study period. All participants underwent neurological examination within one hour after the administration of FUS and were divided into three groups to undergo follow-up anatomical MRI and neurological examination at different time periods, *i.e.*, the same day (*N =* 6), 2 weeks later (*N =* 9), and 4 weeks later (*N =* 4). All of the participants were contacted again by telephone two months after the sonication session and were interviewed to identify the presence of any changes in their mental and physical health status, including experiences of any discomfort associated with the procedures.

Supplementary References

1 Lee, W., Lee, S. D., Park, M. Y., Yang, J. & Yoo, S.-S. Evaluation of polyvinyl alcohol cryogel as an acoustic coupling medium for low-intensity transcranial focused ultrasound. *Int J Imaging Syst Technol* **24**, 332-338 (2014).

2 Yoo, S.-S. *et al.* Focused ultrasound modulates region-specific brain activity. *Neuroimage* **56**, 1267-1275 (2011).

3 Lee, W. *et al.* Image-guided transcranial focused ultrasound stimulates human primary somatosensory cortex. *Sci Rep* **5**, 8743 (2015).

4 Kim, H., Chiu, A., Lee, S. D., Fischer, K. & Yoo, S.-S. Focused ultrasound-mediated non-invasive brain stimulation: examination of sonication parameters. *Brain Stimul* **7**, 748-756 (2014).

5 Lee, W. *et al.* Image-guided focused ultrasound-mediated regional brain stimulation in sheep. *Ultrasound Med Biol* **42**, 459-470 (2016).
